# Supplementary material for: Induced Topological Superconductivity in a BiSbTeSe2-Based Josephson Junction
Source: Nanomaterials (Basel). 2020 Apr 21;10(4):794. doi: 10.3390/nano10040794 (PMC7221935; doi:10.3390/nano10040794)
Supplement: Supplementary file 1 [file nanomaterials-10-00794-s001.pdf]

nanomaterials

MDPI

Article

Induced Topological Superconductivity in a $BiSbTeSe_2$-Based Josephson Junction—Supplementary Information

Bob de Ronde [1], Chuan Li [1], Yingkai Huang [2] and Alexander Brinkman [1]

[1] MESA+ Institute for Nanotechnology, University of Twente, 7522 NB, Enschede,The Netherlands; bobdronde@gmail.com (B.d.R.); chuan.li@utwente.nl (C.L.); a.brinkman@utwente.nl (A.B.)

[2] Van der Waals–Zeeman Institute, IoP, University of Amsterdam, 1098 XH, Amsterdam, The Netherlands; y.huang@uva.nl

* Correspondence: a.brinkman@utwente.nl

Received: 3 March 2020; Accepted: 14 April 2020; Published: 21 April 2020

check for updates

Gating the Fermi Level Close to the Dirac Point

In Figure 4 of the main text, the effect of gating on device 2 is shown. Upon decreasing the top gate voltage, the critical current decreases, while the resistance increases, suggesting the Fermi level is tuned close to the Dirac point. To strengthen this observation, the Fraunhofer patterns at several gate voltages were obtained. Figure S1 shows the Fraunhofer pattern of device to for several top gate voltages. When the top gate voltage is tuned to the negative side, the Fraunhofer pattern becomes less regular. Since the Fraunhofer pattern is a Fourier transform of the current flow through the junction, a regular pattern means a uniform current flow. This leads to the observation that the current flow becomes less regular at negative gate voltages, indicating the presence of disorder. Studies on graphene have shown increased disorder in the form of charge puddles when the Fermi level resides in the vicinity of the Dirac point [1]. Therefore, the irregular Fraunhofer patterns suggest that device 2 is tuned close to the Dirac point at negative gate voltages. This ability to tune the Fermi level close to the Dirac point could prove useful to isolate any zero energy mode for potential applications.

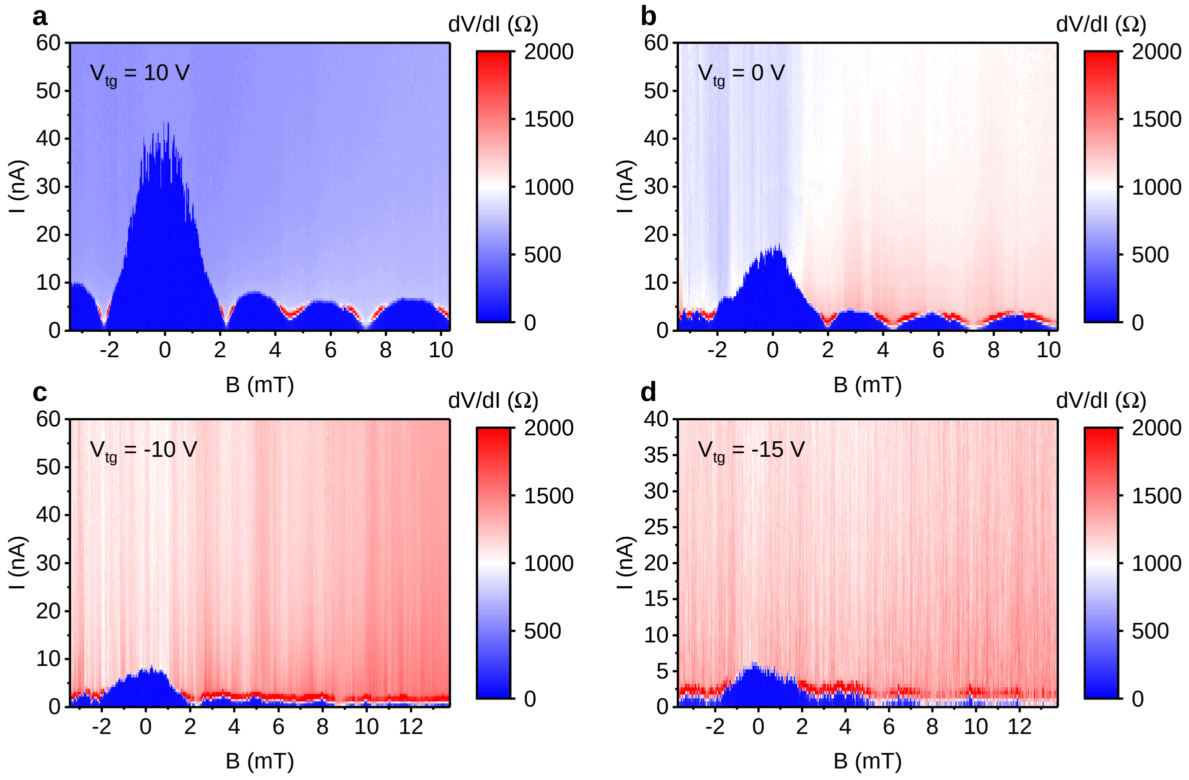

Figure S1. The Fraunhofer pattern of device 2 for a top gate voltage of (**a**) 10 V (**b**) 0 V (**c**) −10 V and (**d**) −15 V. The Fraunhofer patterns become less regular at negative gate voltages, indicating that the Dirac point is close.

Using Temperature to Unveil 4π Periodicity

At low temperature, the re-trapping voltage may be higher than the voltage of some Shapiro steps, obscuring low bias Shapiro steps. As a way to counteract this behaviour, the temperature can be increased. This reduces the gap size and the re-trapping voltage, causing the electrons to occupy a Shapiro step mode. This phenomenon was used to show the robustness of a missing first step in the $BiSbTeSe_2$ junctions. The measurements of a second device, device 1b, similar to device 1a of the main text, but with a length of 250 nm, were used in this analysis.

The radio frequency response at a frequency of 1.85 GHz of device 1b, a 250 nm long junction, at 20 mK and at 1 K is shown in Figure S2a,b, respectively. The analysis of the data is focused on the negative bias voltage side, since the re-trapping current is smaller than the critical current and the voltage was swept from the negative to the positive side. This results in a better visibility of the low order Shapiro steps and thus aids the goal that the increased temperature was attempting to achieve. At 20 mK, the energy gained by sustaining a supercurrent is high enough to obscure the Shapiro steps below the third, as can be seen in Figure S2a. However, the second Shapiro step is fully developed at 1 K, as shown in Figure S2b, without the presence of the first Shapiro step. However, the binning map does not show a value close to zero between zero bias and the second Shapiro step, indicating that there is no sharp transition from zero bias to the Shapiro mode bias. In other words, the gap edges in IV-curve are no longer sharp. The same is observed at a frequency of 3.1 GHz, Figure S2c,d, though a hint of the second step can be distinguished at 20 mK already for this frequency. But there is still no sign of the first step at 1 K. Now for a frequency of 4.7 GHz, Figure S2e,f, the second Shapiro step is fully developed at 20 mK already. However, there is still only a mere hint of a first Shapiro step at 1 K.

This is an unexpected result looking at the energy scale involved in the formation of Shapiro steps. The bias voltage where a Shapiro step appears scales linearly with frequency and with Shapiro step order. Therefore, since 4.7 GHz is more than twice the value of 1.85 GHz, a fully developed first step should be expected at 1 K. This leads to the conclusion that the first Shapiro step is subject to an anomalously strong suppression in the $BiSbTeSe_2$ junctions. This is suggestive of the presence of a Majorana bound state in this system. The same analysis holds for device 1a, of which the measurements at 1.81 GHz, 3.1 GHz and 4.7 Ghz are shown in Figure S3 for both 20 mK and 1 K.

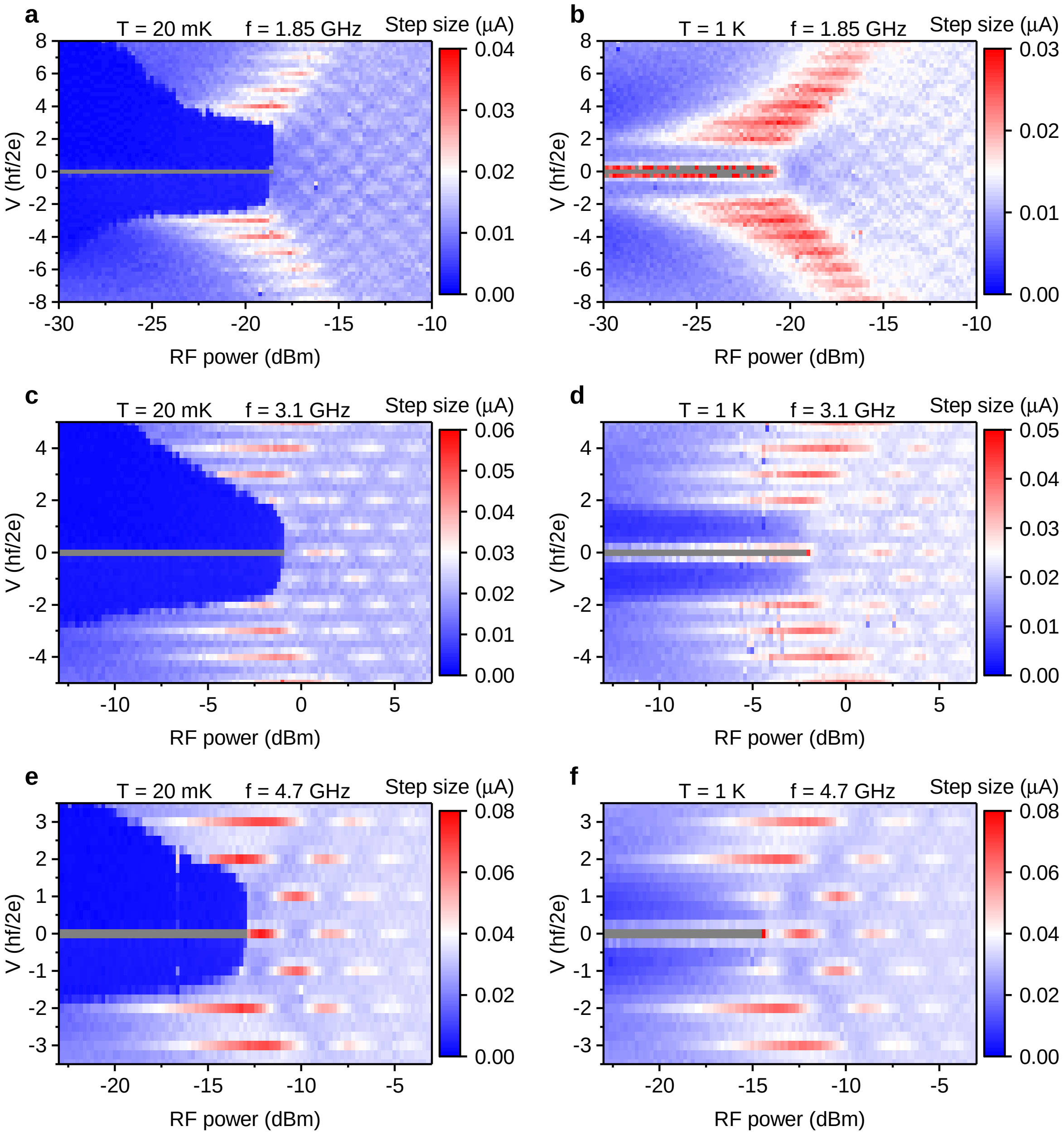

Figure S2. Shapiro step size of junction 1b under radio frequency irradiation at 1.85 GHz at (**a**) 20 mK and at (**b**) 1 K, at 3.1 GHz at (**c**) 20 mK and at (**d**) 1 K, and at 4.7 GHz at (**e**) 20 mK and at (**f**) 1 K. At 1.85 GHz, the second Shapiro step becomes visible at 1 K. At 3.1 GHz, the second Shapiro step is already present to some extent at 20 mK, but it becomes more pronounced at 1 K. At 4.7 GHz, the second Shapiro step is fully developed at 20 mK, but the first Shapiro step remains absent at 1 K.

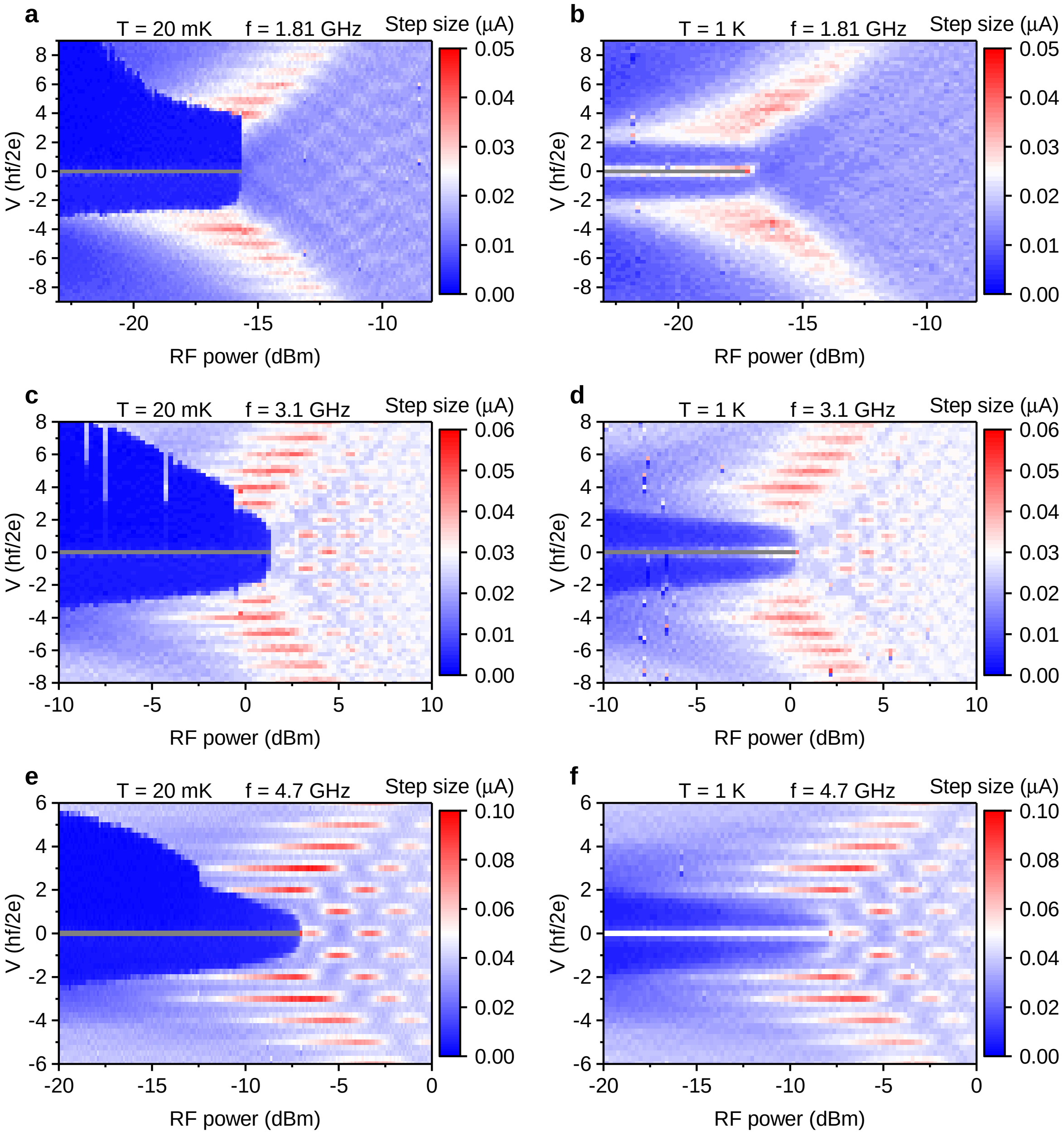

Figure S3. Shapiro step size of junction 1a under radio frequency irradiation at 1.81 GHz at (**a**) 20 mK and at (**b**) 1 K, at 3.1 GHz at (**c**) 20 mK and at (**d**) 1 K, and at 4.7 GHz at (**e**) 20 mK and at (**f**) 1 K. At 1.81 GHz, the second Shapiro step becomes visible at 1 K. At 3.1 GHz, the second Shapiro step is already present to some extent at 20 mK, but it becomes more pronounced at 1 K. At 4.7 GHz, the second Shapiro step is fully developed at 20 mK, but the first Shapiro step remains absent at 1 K.

References

1. Samaddar, S.; Yudhistira, I.; Adam, S.; Courtois, H.; Winkelmann, C.B. Charge Puddles in Graphene near the Dirac Point. *Phys. Rev. Lett.* **2016**, *116*, 126804.

© 2020 by the authors. Licensee MDPI, Basel, Switzerland. This article is an open access article distributed under the terms and conditions of the Creative Commons Attribution (CC BY) license (http://creativecommons.org/licenses/by/4.0/).
